# Nasal microbiota clusters associate with inflammatory response, viral load, and symptom severity in experimental rhinovirus challenge

**DOI:** 10.1038/s41598-018-29793-w

**Published:** 2018-07-30

**Authors:** Markus J. Lehtinen, Ashley A. Hibberd, Sofia Männikkö, Nicolas Yeung, Tommi Kauko, Sofia Forssten, Liisa Lehtoranta, Sampo J. Lahtinen, Buffy Stahl, Anna Lyra, Ronald B. Turner

**Affiliations:** 1 Global Health & Nutrition Science, DuPont Nutrition and Health, Sokeritehtaantie 20, FI-02460 Kantvik, Finland; 2 Genomics and Microbiome Science, DuPont Nutrition and Health, 3329 Agriculture Drive, Madison, WI 53716 USA; 3 4Pharma Ltd., ElectroCity, Tykistökatu 4D, FI-20520 Turku, Finland; 4 University of Virginia, School of Medicine, P.O. Box 800386, Barringer Building, Room 4441, Hospital Drive, Charlottesville, VA 22908 USA

## Abstract

The role of nasal and fecal microbiota in viral respiratory infections has not been established. We collected nasal swabs and washes, and fecal samples in a clinical study assessing the effect of probiotic *Bifidobacterium animalis* subsp. *lactis* Bl-04 on experimental rhinovirus infection. The nasal and fecal microbiota were characterized by 16S rRNA gene sequencing. The resulting data were compared with nasal inflammatory marker concentrations, viral load, and clinical symptoms. By using unsupervised clustering, the nasal microbiota divided into six clusters. The clusters predominant of *Staphylococcus, Corynebacterium/Alloiococcus, Moraxella, and Pseudomonadaceae*/Mixed had characteristic inflammatory marker and viral load profiles in nasal washes. The nasal microbiota clusters of subjects before the infection associated with the severity of clinical cold symptoms during rhinovirus infection. Rhinovirus infection and probiotic intervention did not significantly alter the composition of nasal or fecal microbiota. Our results suggest that nasal microbiota may influence the virus load, host innate immune response, and clinical symptoms during rhinovirus infection, however, further studies are needed.

## Introduction

Rhinoviruses are an important cause of respiratory illness. Adults have 1–2 rhinovirus infections each year and approximately 60% of these infections are associated with symptoms^1,2^. The symptoms of rhinovirus infections are closely associated with the innate inflammatory response to the virus^3^. The concentration of pro-inflammatory cytokines in nasal lavage has a modest correlation with symptom severity, and expression of innate immunity-associated genes reliably distinguishes symptomatic from asymptomatic infections^4–6^. These observations suggest that modulation of the innate host response is a potential target for intervention in these illnesses.

There is substantial evidence that the gut microbiome can influence innate immune responses and alterations in the gut microbiota in animal models have been linked to changes in the response to viral respiratory infections^7–10^. Although human data are limited, a study of bronchiolitis found that infants with a *Bacteroides*-dominant fecal microbiota profile were more likely to develop bronchiolitis than those with an *Enterobacter/Veillonella*-dominant profile, suggesting that modulation of human host responses by the fecal microbiome may have clinically relevant impacts on respiratory disease^11^. Much less is known about interactions among the nasal microbiota, respiratory host responses, and viral infection. Firmicutes and Actinobacteria have been identified as the predominant phyla detected in the nasal microbiota^12,13^. Specimens collected by nasal lavage or nasopharyngeal swabs also contained bacteria from phylum Proteobacteria consistent with the microbiota characteristics of the oropharynx. Attempts to correlate the microbial profile at the phylum level with clinical characteristics during viral respiratory infection have been generally unsuccessful. In contrast, there is a suggestion that changes in the microbiota during infection or associations between the microbial profile and clinical characteristics can be detected when the nasopharyngeal microbiota is characterized at the genus level^14–16^. Furthermore, a study in children showed that nasal microbiota clusters rich in *Haemophilus influenzae* or *Streptococcus* spp. were associated not only with respiratory syncytial virus (RSV) infection but also with overexpression of inflammatory markers^17^.

Meta-analyses suggest that probiotics could reduce the risk and duration of acute upper respiratory tract infections^18^. These effects are likely driven by strain-specific effects on immune function^19^. Studies in mice show that direct application of probiotics into nostrils modulates the local immune response against viral infections^20,21^ and consumption of probiotics by humans changes the immune function in the small intestine^22^. However, clinical studies assessing the impact of probiotics on the intestinal or upper respiratory tract microbiota in the context of host response and illness are limited.

A randomized controlled trial of *Bifidobacterium animalis* subsp. *lactis* Bl-04 (Bl-04) found that Bl-04 consumption was associated with reduced risk of upper respiratory illness episodes over a 5-month winter period in adults^23^. In an experimental rhinovirus challenge model, Bl-04 modulated the inflammatory response and viral load in nasal washes, but did not have an impact on symptom severity scores^24^. We hypothesized that nasal and intestinal microbiotas could have an effect on rhinovirus infection and host inflammatory response, and on the other hand that oral probiotic administration or rhinovirus infection could potentially impact intestinal or nasal microbiotas. Thus, nasal swabs and fecal samples collected during the latter study were used to explore the interactions among the nasal and fecal host microbiota, the administration of probiotic, and the clinical results of the viral infection under controlled conditions.

## Results

### Nasal microbiota grouped into six clusters

**Figure 1:**
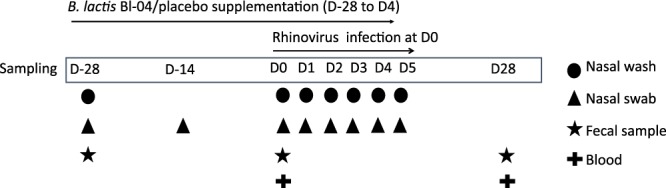
Study outline and sampling set-up. Subjects were enrolled from three separate cohorts to receive either Bl-04 (2 × 10^9^ CFU/day) or placebo for 28 days prior to being challenged with an experimental rhinovirus. The participants continued investigational product intake for 4 days post-challenge. On study day (D) 0, subjects were inoculated with rhinovirus type A39 (FDA-BB-IND #12934) (100 TCID_50_, split between both nostrils). On D1–D5, participants returned to the study site for assessment of infection symptoms assessed by WURSS-21, collection of nasal lavage for inflammatory marker analyses, and quantitative rhinovirus culture. Volunteers returned a final time at D28 for collection of convalescent serum for antibody to rhinovirus type A39.

**Figure 2:**
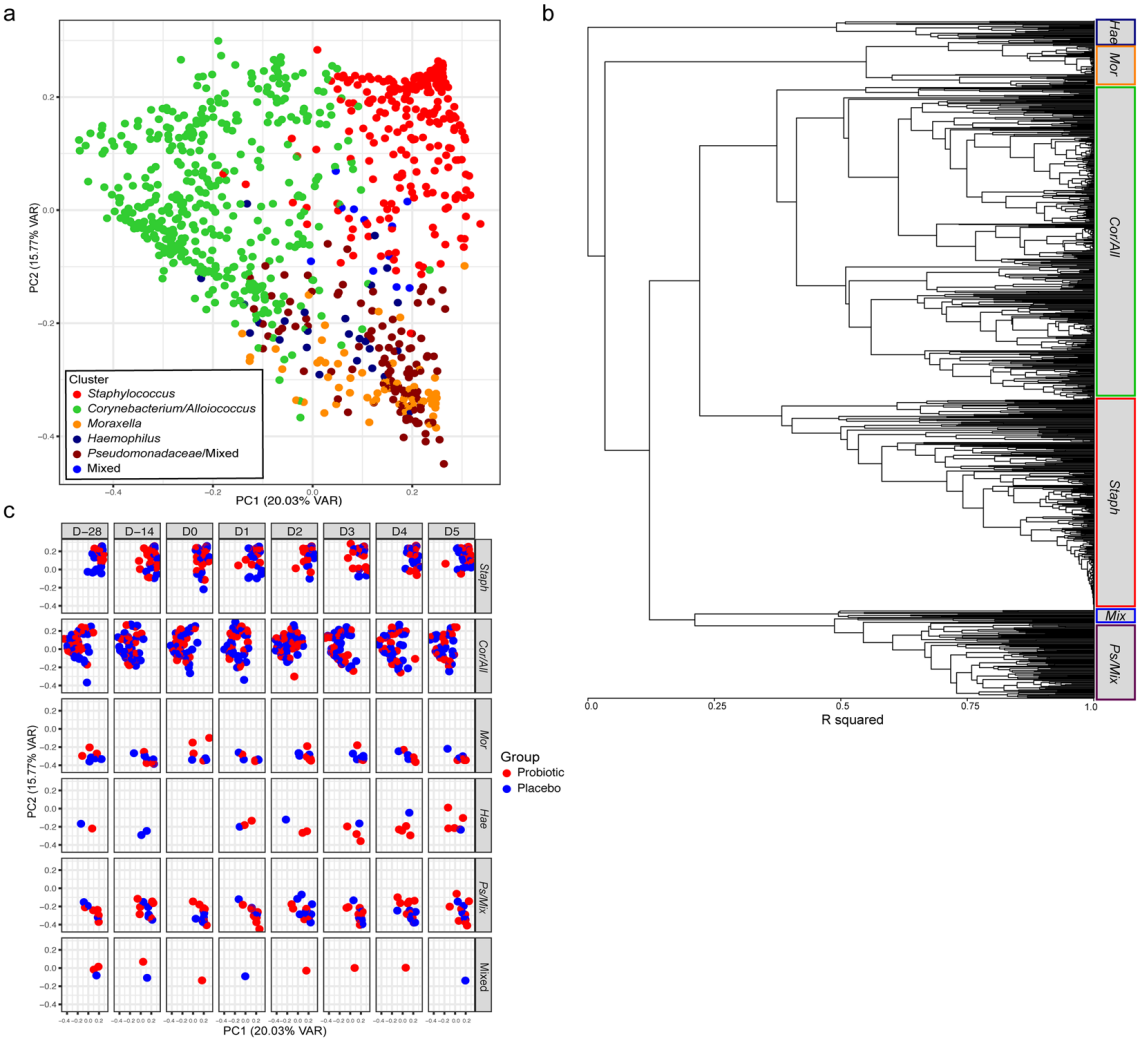
Clustering of nasal microbiota samples. (**a**) Principal coordinates plot showing clustering independent of time point and treatment. (**b**) Unweighted pair-group method (UPGMA) clustering of samples (**c**) Principal coordinates plot of sample distribution in different time points and treatment groups.

**Figure 3:**
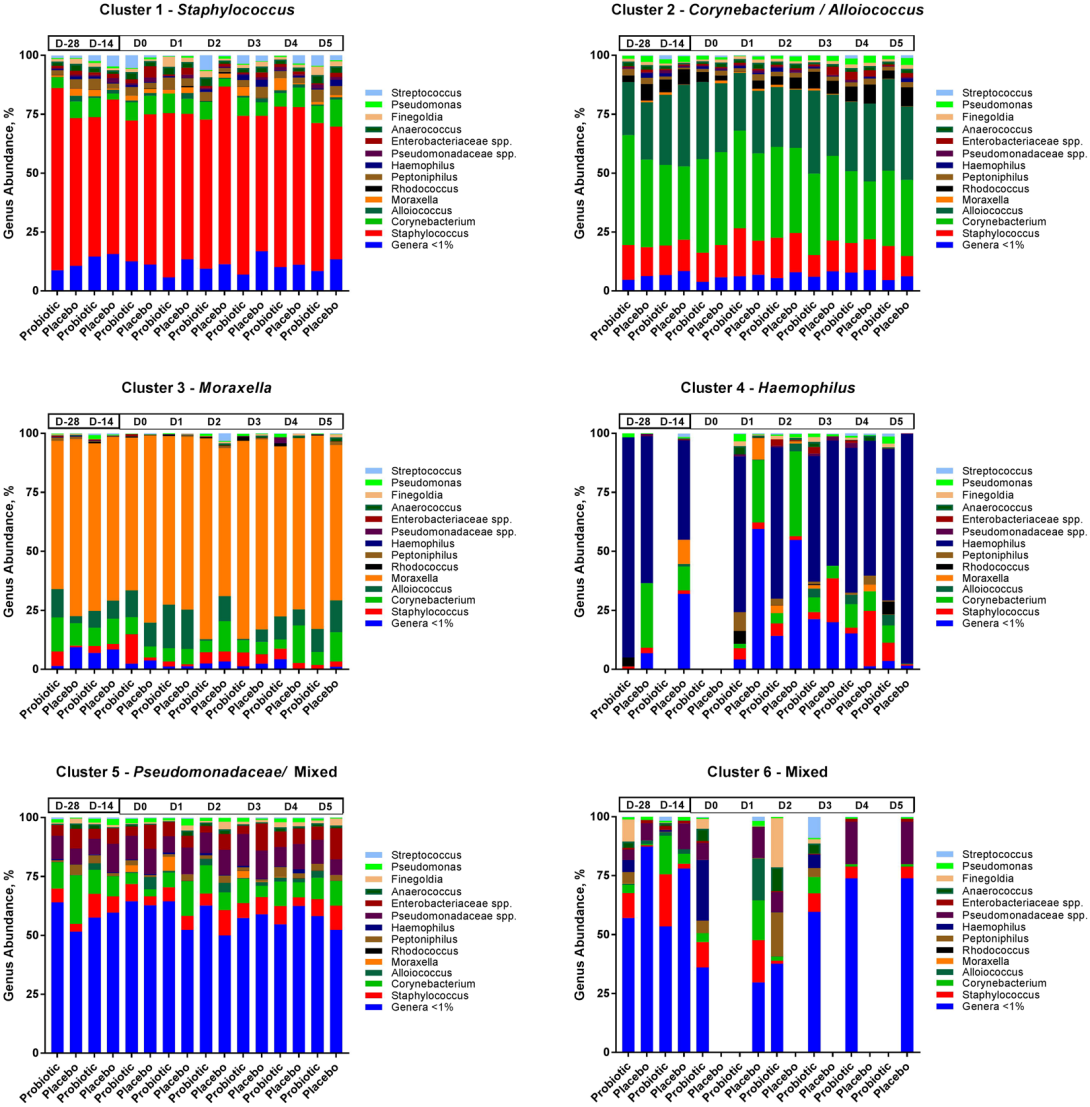
Relative abundance (%) of bacterial genera in the nasal microbiotas of six clusters in the two study groups over study time. The number of samples for each time and treatment point is shown in Supplementary Table 1.

**Figure 4:**
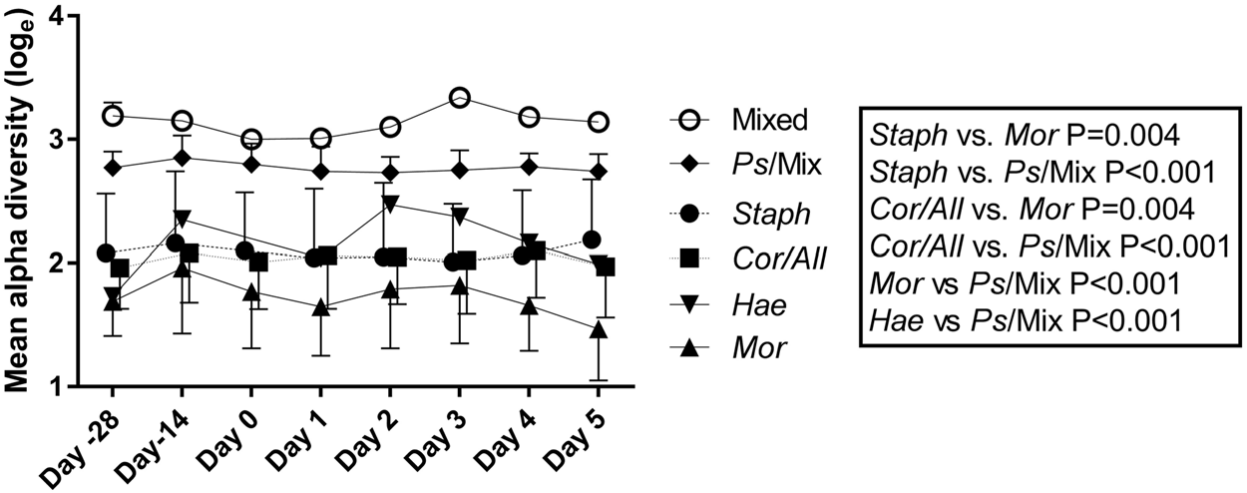
Mean alpha diversities of the clusters over time. *Staph* vs. *Ps*/Mix estimated mean difference (EMD) [log] = −0.35, *Cor/All* vs. *Ps*/Mix EMD [log] = −0.35, *Mor* vs. *Ps*/Mix EMD [log] = −0.54, *Hae* vs. *Ps*/Mix EMD [log] = −0.37, *Staph* vs. *Mor* EMD [log] = 0.19, and *Cor/All* vs. *Mor* EMD [log] = 0.19. RM ANCOVA, all p-values are unadjusted. Over time total N: Mixed N = 11, *Ps*/Mix N = 110, *Staph* N = 288, *Cor/All* N = 429, *Hae* N = 25, and *Mor* N = 66.

Nasal swabs were collected pre- and post-supplementation and during the infection for sequencing (Fig. 1). The results showed that the most abundant phyla in the nasal microbiota were Firmicutes (51%), Actinobacteria (29%), and Proteobacteria (19%) and the most abundant genera were *Staphylococcus* (30%), *Corynebacterium* (25%), *Alloiococcus* (13%), and *Moraxella* (5.9%). The nasal microbial taxa were tested for the presence of microbiota subtypes by using unsupervised clustering. The analysis showed that the samples grouped into six clusters that were named according to the dominant bacterial genera in the samples: *Staphylococcus* (*Staph*)*, Corynebacterium/Alloiococcus* (*Cor/All*)*, Moraxella* (*Mor*)*, Haemophilus* (*Hae*)*, Pseudomonadaceae/*Mixed (*Ps*/Mix), and Mixed. Sample grouping by the 6 clusters explained 49% of the variation by PCoA (adonis R^2^ = 0.4899; P < 0.001) (Fig. 2a), and sample designation to clusters by UPGMA are shown in Fig. 2b. The *Mor*, *Hae*, and *Ps*/Mix clusters had clear dominance of Proteobacteria, whereas *Staph* cluster was dominated by Firmicutes and *Cor/All* cluster by Firmicutes and Actinobacteria (Supplementary Fig. 1). At the pre-intervention baseline D-28 (n = 122), the most frequent clusters were the *Cor/All* (51%) and *Staph* (31%), whereas *Mor* (7.4%)*, Ps/*Mix (6.6%), Mixed (2.5%) and *Hae* (1.6%) clusters were less common (Supplementary Table 1). Some subjects switched between two to three cluster types over the study time, while others were consistently having one cluster type (Supplementary Fig. 2). The rhinovirus infection or probiotic intervention did not significantly affect the distribution of the clusters (P > 0.05, logistic regression GEE model for the 2 largest clusters) (Fig. 2c and Supplementary Table 1) or the relative abundances of the taxa (Fig. 3). Alpha-diversity was overall found to be significantly different between the clusters (P < 0.0001). Alpha-diversity was significantly higher in *Ps*/Mix cluster compared to other clusters (Fig. 4) and the alpha-diversities in *Staph* and *Cor/All* clusters were significantly higher compared to *Mor* cluster (Fig. 4). Alpha-diversities were not significantly different between the timepoints within the clusters (P > 0.05, RM ANCOVA).

We further tested the effect of the cohort on cluster frequencies at D0 and over all timepoints and found that at D0 *Ps*/Mix cluster was only found in cohort 3 and there were no clusters assigned to *Hae* (Supplementary Table 2). Further, over all time points the *Ps*/Mix cluster was found almost exclusively in the cohort 3. Cohort 3 samples were collected in autumn whereas samples from cohorts 1 and 2 in spring. To assess robustness of the results, the analysis models discussed below were computed separately for each cohort taking into account that comparisons between clusters could only be done for clusters present within each cohort. Some variability was observed in the results between the cohorts, but in general the effects were not in contradiction. Cluster effect might be partly confounded with cohort, but cannot be fully investigated with the current sample size for all clusters. Thus, we pooled the data from all 3 cohorts at D0 and investigated the effect of the pre-infection nasal microbiota on rhinovirus infection.

### Nasal microbiota clusters associate with changes in host inflammatory response

**Figure 5:**
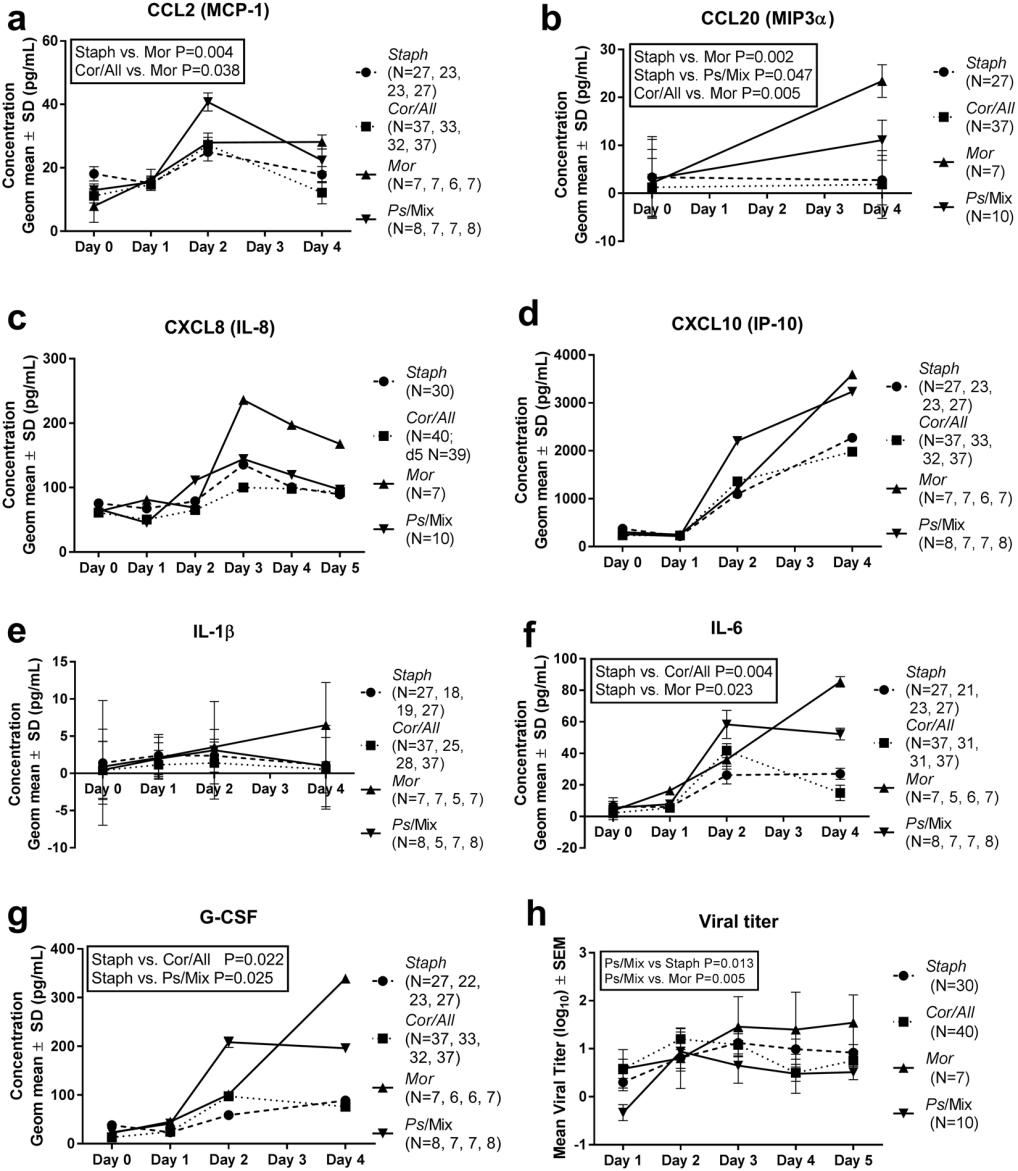
Inflammatory marker response and viral titer during rhinovirus infection. Inflammatory marker concentrations (**a**–**g**) were determined from nasal washes D0–D5 by ELISA. Geometric mean values from D0 to D5 are shown for *Staph*, *Cor/All*, *Mor*, and *Ps*/Mix clusters. Viral titer (**h**) was analyzed from nasal washes D1–D5. Statistical testing for the change in inflammatory markers (**a**–**g**) from D0 and for viral titer (**h**) by cluster was assessed with RM ANCOVA.

We investigated whether the clusters associate with changes in nasal wash inflammatory marker concentrations and viral load at D0–D5 by examining subjects based on their D0 cluster. Only the four clusters *Staph*, *Cor/All*, *Mor*, and *Ps*/Mix with adequate number of samples for statistics were analyzed (Supplementary Table 1). Overall the D0 cluster had a statistically significant effect on change from D0 in G-CSF, CCL20 (MIP3-α), IL-6 and CCL2 (MCP-1) concentrations (P = 0.045, P = 0.005, P = 0.016, P = 0.035, respectively) (Fig. 5). The results showed that the increase from D0 in concentration of CCL class chemokines CCL2 (MCP-1) and CCL20 (MIP3-α) was higher in *Mor* cluster compared to *Staph* and *Cor/All* clusters (Fig. 5a,b). In addition, *Staph* cluster had a lower change in concentration of CCL20 (MIP3-α) from D0 to D4 compared to *Ps*/Mix cluster. CXCL class chemokines CXCL8 (IL-8) and CXCL10 (IP-10) as well as IL-1β had a similar change in response across the clusters (Fig. 5c–e). In the *Staph* cluster changes in the IL-6 levels were lower on average compared to *Cor/All* and *Mor* clusters (Fig. 5f). Also, changes from D0 in G-CSF levels in subjects having *Staph* cluster were smaller compared to *Cor/All* and *Ps*/Mix clusters (Fig. 5g). In general, the *Staph* cluster had the smallest and *Mor* cluster the largest changes from D0 in concentrations of inflammatory markers in the nasal washes during the rhinovirus infection.

### Nasal microbiota clusters associate with differences in viral load

Viral infection was documented by isolation of virus in cell culture or seroconversion to the study virus in 127 (84%) of the 152 subjects in this study. Differences in the viral load based on the D0 clusters was tested. Overall the D0 cluster effect was found to be statistically significant (P = 0.025). The *Ps*/Mix cluster had lower viral titers over the 5 days of infection compared to *Staph* and *Mor* clusters (Fig. 5h). There was no difference in the convalescent antibody titer to the virus between the subjects divided by D0 clusters (P = 0.84, Kruskal-Wallis test).

### Nasal microbiota associates with symptom severity

**Figure 6:**
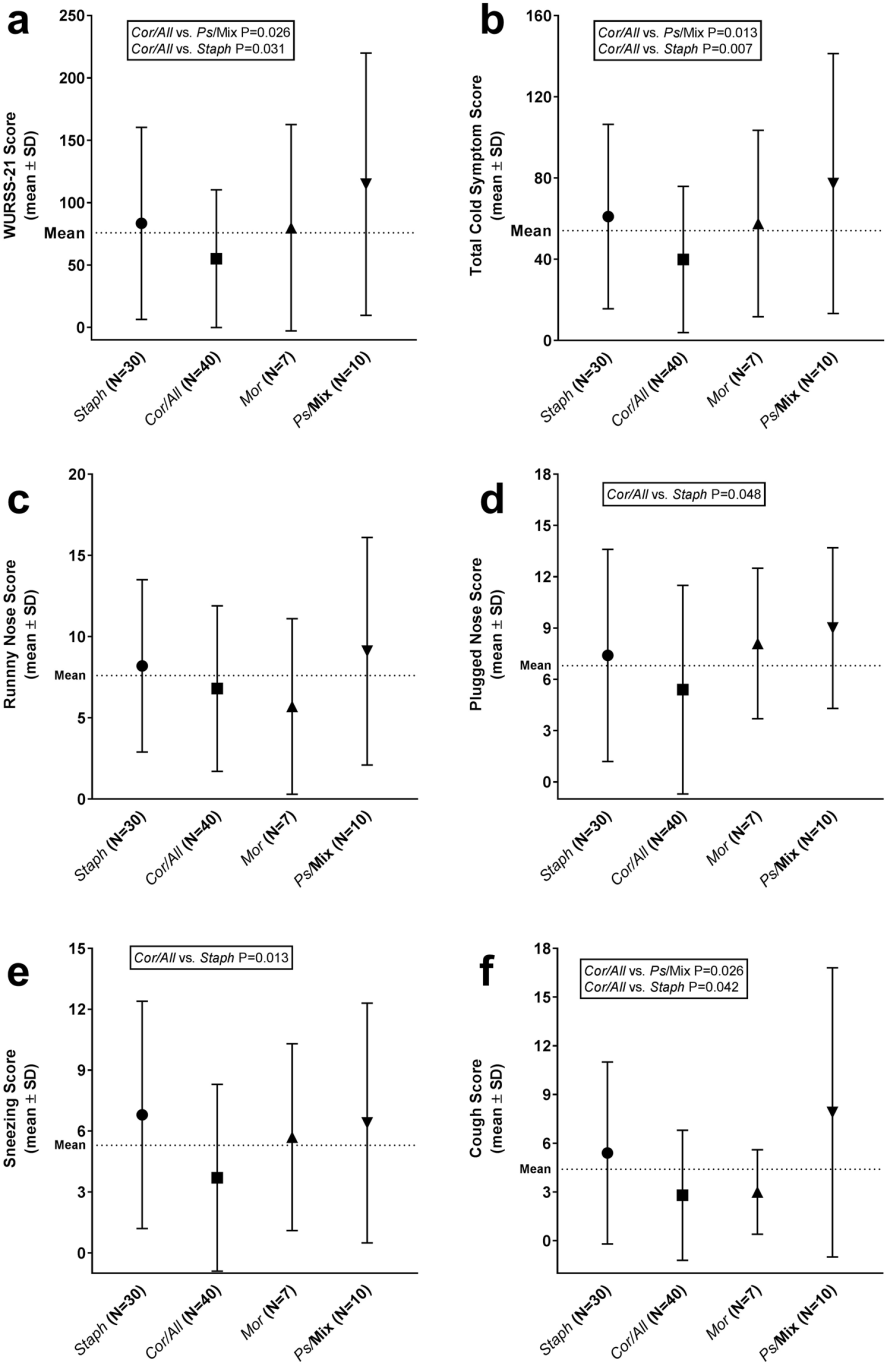
Nasal microbiota cluster and clinical symptoms during rhinovirus infection. The symptom scores for clusters *Staph*, *Cor/All*, *Mor*, and *Ps*/Mix were evaluated over D1–D5 by using (**a**) WURSS-21 questionnaire. Scores for sub-category (**b**) Total Cold Symptoms, and for individual symptoms (**c**) Runny Nose, (**d**) Plugged Nose, (**e**) Sneezing, and (**f**) Cough in WURSS-21 are shown separately. The statistical testing was performed with negative binomial (GEE) model.

The cold symptom scores collected with the 21 item Wisconsin Upper Respiratory Symptom Score (WURSS-21) questionnaire over D1–D5 were compared between the subjects assigned to four clusters *Staph*, *Cor/All*, *Mor*, and *Ps*/Mix at D0. Overall the D0 cluster effect on total symptom scores did not reach statistical significance (P = 0.06). Anyhow, few differences between the clusters were detected. WURSS-21 score, including functional and symptom scores, was lower in *Cor/All* cluster compared to *Staph* (P = 0.031) and *Ps*/Mix clusters (P = 0.026) (Fig. 6a). With total Cold Symptom scores (runny nose, plugged nose, sneezing, sore throat, scratchy throat, cough, hoarseness, head congestion, and feeling tired) a significant D0 cluster effect was found (P = 0.011). The scores were lower in *Cor/All* cluster compared to *Staph* (P = 0.007) and *Ps*/Mix clusters (P = 0.013) (Fig. 6b). When individual symptoms were analyzed, the D0 cluster effect was found not significant (P > 0.1) in all analysis models. There was no difference between the clusters in the runny nose symptom scores (Fig. 6c), while the *Cor/All* cluster had lower scores in nasal obstruction, sneezing, and cough compared to *Staph* cluster (P = 0.048, P = 0.013, P = 0.042, respectively) (Fig. 6d–f). *Ps*/Mix cluster had higher cough symptom score than *Cor/All* cluster (P = 0.026)

We further tested if different symptoms were associated with changes in nasal microbiota alpha diversity. Overall, severity of rhinorrhea was found to be associated with changes in alpha diversity (P = 0.014). The subjects who had more severe rhinorrhea (runny nose score over the median of 7) also had a significantly larger increase in alpha-diversity following the viral challenge (D0) over time compared to the subjects with less severe or no rhinorrhea (high vs. low rhinorrhea symptoms; geometric mean ratio 1.190, 95% CI 1.036–1.366 RM-ANCOVA). No significant changes in alpha diversity between other symptom score groups were detected.

### Bl-04 intervention and rhinovirus infection had minimal effect on nasal microbiota

The presence of Bl-04 in the nasal cavity of 52 subjects was evaluated by qPCR (Table 1). All the samples in the placebo group tested negative for Bl-04 except for one D0 sample. In the probiotic group, Bl-04 was not detected at D-28, but was found after supplementation in <18% of samples and during the infection in <10% of the samples. By sequencing, genus *Bifidobacterium* was detected at a significantly greater abundance in the probiotic group after supplementation period at D0, however, after multiple testing correction the effect was non-significant (probiotic vs. placebo: 0.2217% vs. 0.0198%; P = 0.0034, FDR P = 0.169, Kruskal-Wallis test). No differences were detected in phylum- or genus level-taxa abundances between probiotic and placebo groups for any study day (data not shown, FDR P > 0.1). Neither the probiotic intervention nor rhinovirus infection had an effect on the alpha diversity (Supplementary Table 3) or beta diversity with either unweighted or weighted UniFrac (Supplementary Fig. 3 and Supplementary Table 4) indicating that nasal microbiota remained stable during the rhinovirus infection in both intervention groups.

**Table 1:** Number and distribution of nasal and fecal samples between study groups at different sampling times and number of Bl-04 positive samples by qPCR (qPCR+).

Sampling time	Nasal 16S rRNA sequencing		Nasal qPCR		Fecal qPCR and 16S rRNA sequencing	
	Probiotic N	Placebo N	Probiotic N (qPCR+)	Placebo N (qPCR+)	Probiotic N (qPCR+)	Placebo N (qPCR+)
D-28	60	62	25 (0)	33 (0)	70 (8)	72 (3)
D-14	54	59	33 (6)	31 (0)		
D0	60	63	32 (4)	32 (1)	72 (50)	79 (0)
D1	57	60	31 (3)	32 (0)		
D2	58	59	31 (2)	33 (0)		
D3	57	56	31 (0)	33 (0)		
D4	57	57	30 (2)	34 (0)		
D5	58	54	29 (1)	32 (0)		
D28					73 (6)	78 (3)
Total	461	470	242 (18)	260 (1)	215 (64)	229 (6)

### Bl-04 intervention had no effect on fecal microbiota

Fecal samples were collected at day (D) −28, D0, and D28 to analyze the effect of the Bl-04 on the fecal microbiota (Fig. 1). qPCR analyses showed that after 4 weeks of supplementation and pre-infection, Bl-04 was detected only in the probiotic group (Table 1). The 16S rRNA gene sequencing analyses did not show any effect of the probiotic on the fecal microbiota composition or on alpha diversity (Supplementary Fig. 4 and Supplementary Table 5). Additionally, there were no significant differences according to treatment for beta diversity (Supplementary Table 6) or individual taxa. The fecal microbiota sequence data at D0 did not fall into clear clusters and was not subject to further analysis in respect to biomarkers, viral load, or symptoms.

## Discussion

We found that nasal microbiotas of healthy adults in our study cohort grouped into six distinct clusters *Staph*, *Cor/All*, *Mor*, *Hae*, *Ps*/Mix, and Mixed, based on the predominant bacterial genera in the nasal microflora. The microbiota clusters appear to associate with different inflammatory response, viral titer, and symptom severity profiles in rhinovirus infection. Our study is aligned with the previous findings suggesting that the microbiota in the nose has seasonal variation. We further found that rhinovirus infection did not have a major impact on the nasal microbiota. Furthermore, oral probiotic consumption did not have an effect on nasal or fecal microbiota composition. The results of this study raise the possibility that the modulation of the nasal microbiota, perhaps by intranasal administration of probiotics, might provide an approach to intervention in rhinovirus-associated illness or other viral infections.

In our study cohort of healthy young adults, the major phyla in the nasal microbiota were Firmicutes, Actinobacteria, and Proteobacteria, whereas the major genera were *Staphylococcus*, *Alloiococcus*, *Corynebacterium*, and *Moraxella*. The result is well-aligned with the previously published smaller studies in adults^12,13,25^. We applied a clustering algorithm to describe our data and showed that the nasal microbiota samples fall into six clusters *Staph*, *Cor/All*, *Mor*, *Hae*, *Ps*/Mix, and Mixed that were named according to predominant bacterial genera in each (Fig. 2a). Of the clusters identified, the *Cor/All* and *Staph* clusters were the most common in the study cohort of our healthy adults. The dominant genera within clusters consisted of several OTUs that varied greatly in presence and abundance across individuals rather than single dominant OTUs. The designation of microbiota clusters represented our data well and described 49% of the variation observed; however, a limitation of unsupervised clustering is the possibility that clusters could change when applied to other cohorts. Other studies have used clustering approaches to describe the nasal microbiota, and similar dominant bacteria clusters (15% relative abundance for *Staphylococcus* and *Corynebacterium*) were reported in posterior nasopharynx swabs from have been reported in healthy adults by swabs from posterior nasopharynx^25^. In children, however, the dominant clusters seem to differ compared to adults with almost equal occurrence of *Moraxella*, *Streptococcus*, *Staphylococcus*, *Corynebacterium*, and *Haemophilus* rich clusters^17^. We further found that *Ps*/Mix cluster was almost exclusively discovered in the autumn cohort. Previous studies have shown that risk for *Pseudomonas aeruginosa* infection is higher in autumn and winter months^26–28^, suggesting that perhaps changes in environment favor enrichment of Pseudomonas species in the nasal microbiota.

We further tested the association of the four nasal microbiota clusters with the inflammatory response to rhinovirus infection. The analysis was limited to clusters with adequate number of nasal wash samples at D0. Subjects with *Staph*, *Cor/All*, *Mor*, and *Ps*/Mix clusters were found to have differences in CCL2, CCL20, IL-6, and G-CSF responses during the infection (Fig. 5a–h). In general, the change in the concentrations of these inflammatory mediators from D0 tended to be higher for the *Mor* and *Ps*/Mix clusters and lower for the *Staph* and *Cor/All* clusters. The target receptors for CCL2 (CCR2) and CCL20 (CCR6) are found mainly on monocytes, immature DCs, B-cells and activated T-cells^29^. On the other hand, the nasal microbiota clusters had no impact on (i) CXCL8 response targeting CXCR1 and 2 on neutrophils, or (ii) on CXCL10 response targeting CXCR3A and B on natural killer and T cells^29^. The differences in chemokine profiles thus suggest that nasal microbiota may influence the population of immune cells recruited at the site of rhinovirus infection. In addition, differences in IL-6 and G-CSF induction imply that early inflammatory events and hematopoiesis may be induced differently by the nasal microbiota clusters. A similar effect of the microbiota on cytokine signatures has been described in the intestinal environment^30^. Further, it is reasonable to speculate that synergistic stimuli by nasal microbiota and rhinovirus infection drive the immune response as demonstrated in lung epithelial cells^31^.

We further analyzed the association of the nasal microbiota clusters at the time of the infection with the rhinovirus load in nasal washes. Subjects with *Ps*/Mix cluster had significantly lower viral load than those subjects with *Mor* or *Staph* predominant microbiota types (Fig. 5g). Thus, microbiota at the time of infection may influence subsequent rhinovirus viral load. The results also suggest that high inflammatory response does not lead to low viral load as the subjects with *Ps*/Mix and *Mor* clusters had both relatively high inflammatory cytokine response and only *Ps*/Mix cluster had low viral load. In mice, it has been shown that commensal bacterium from human nasal microbiota, *Corynebacterium pseudodiphtheriticum* was able to reduce respiratory RSV loads in lungs when applied nasally^32^. The modulation of human nasal microbiota in respiratory infections warrants further investigation.

The pre-infection nasal microbiota type was also associated with effects on symptom severity. Subjects with the *Cor/All* cluster had significantly lower cold symptom scores than the subjects with the *Staph* or *Ps*/Mix clusters. The individual symptoms sneezing, plugged nose, and cough followed a similar pattern. The results indicate that nasal microbiota has a role in the presentation of viral upper respiratory illness symptoms. It has been shown that the severity of illness is modestly correlated with the innate inflammatory CXCL8 and CXCL10 response to the virus^4,33^, however the nasal lavage concentrations of these cytokines were similar between the clusters. We found no apparent correlation between the inflammatory response, viral load and symptoms in this study. Others have also attempted to assess associations between microbial profiles and disease severity. Young infants with an RSV infection had a greater abundance of *Streptococcus*, *Moraxella*, and *Haemophilus* than healthy infants^16^. In subsequent studies, the *Haemophilus*-dominant profile was associated with more severe illness^15,17^ and *Haemophilus* and *Streptococcus* dominant clusters were associated with higher inflammatory response in blood samples compared to *Corynebacterium*, *Moraxella*, and *S. aureus* clusters. Interpretation of the findings is complicated by the fact that different viral pathogens (rhinovirus or RSV) appear to be associated with different microbial profiles in the nasopharyngeal samples^34,35^.

The results of our study suggest that rhinovirus infection did not have a major impact on the microbiota, although we found an association between rhinorrhea severity and increased alpha-diversity after the virus challenge. Causality was not established in this study, but it seems possible that the increase in alpha-diversity was a result of the increased rhinorrhea rather than *vice versa*^36^. Previously the interactions between the nasal or nasopharyngeal microbiota and viral respiratory infection have been explored in a limited number of human studies. A convenience sample of patients with H1N1 type influenza found that during infection the microbial profile was similar to that described in healthy individuals and the profile had no correlation with the demographic characteristics of the patients^37^. Two small studies using the experimental rhinovirus model^38,39^ reported no detectable variation in the composition of the microbiome in relation to time, illness, or infection status.

The inclusion of probiotic strain Bl-04 in our study permitted a controlled evaluation of the interaction of the probiotic with the nasal and fecal microbiota. We did not observe any statistically significant impact by the probiotic intervention on the microbiota in the study subjects, however, we detected Bl-04 more abundantly from nasal swabs and fecal samples in the probiotic group (Table 1) suggesting that oral consumption may lead to exposure of nasal mucosa to probiotics. In a previous report, we have described a modest but significant effect of Bl-04 on the innate inflammatory response and on decreasing viral shedding and viral load in nasal washes following infection^24^. The lack of a detectable effect of Bl-04 on the nasal microbiota suggests that the inflammatory and viral shedding effects are not mediated through significant changes in the nasal or fecal microbiota, but perhaps rather driven by direct contact of the probiotic with the mucosal immune system. Clear effects by the *Lactobacillus* probiotics on the small intestinal mucosal immune system has been shown in a human study^22^. In an animal model of viral infection, oral as well as intranasal administration of probiotics have stimulated a mild inflammatory response and moderated the severity of subsequent illness^21,40^. Whether intranasal administration of probiotic to the human host would amplify the effects seen following oral administration remains to be determined.

The interaction between the upper respiratory microbiota and rhinovirus infection is largely unexplored. We demonstrated that the baseline nasal microbiota clusters were associated with subsequent nasal inflammatory response, viral titer, and symptom severity in rhinovirus infection. Given the exploratory nature of our study, further investigations are needed to confirm the relationship between the nasal microbiota, host response and viral load. Neither the probiotic nor the rhinovirus infection had any clear effect on the composition of the nasal microbiome. Our results suggest that interventions targeting the composition of the nasal microbiota could be beneficial in upper respiratory tract infections.

## Methods

### Study set-up and sampling

The volunteers included in this study were 152 healthy young adults (Supplementary Table 7) who were challenged with rhinovirus type 39 as part of randomized, double blind, placebo controlled clinical trial investigating the effects of probiotic Bl-04 on the immune response to rhinovirus at the University of Virginia (ClinicalTrials.gov NCT01669603)^24^. This study was reviewed and approved by the by the Institutional Review Board for Health Sciences Research at the University of Virginia. Written informed consent was obtained prior to study participation from all participants. The study was conducted in compliance with Good Clinical Practices and in accordance with the Declaration of Helsinki. Subjects were compensated for participation. The scheme for the intervention, viral challenge, and sampling is shown in Fig. 1.

### DNA extraction, sequencing and qPCR

For microbiota analyses nasal swabs and stool samples were collected (Table 1). A nylon swab was moistened in sterile saline and then inserted through the nares and passed beneath the inferior turbinate until resistance was met. The swab was then gently rotated, removed, and then the other side of the nose was swabbed in the same way with the same swab. The swab specimens were stored in cryovials at −70 °C. The fecal samples were defecated on the same day, kept at 4 °C before and during transport to the clinic and immediately frozen at −70 °C for the analyses.

Microbial DNA was extracted and purified with an automated MagMAX™ Sample Preparation System (Life Technologies, Halle, Belgium), by using the MagMAX™ Total Nucleic Acid Isolation Kit, AM1840 (ThermoFisher Scientific OY, Vantaa, Finland) according to the manufacturer’s instructions with some modifications. DNA quality and quantity of fecal samples were adequate to perform sequencing and qPCR on all available samples (n = 444). Out of 1207 nasal samples only 931 had adequate DNA quality and quantity for sequencing. Due to low yield of DNA from nasal swabs only 502 samples were analyzed for the presence of Bl-04 by qPCR. Bl-04 was quantified with strain specific primers using standard qPCR technology and the microbiota composition was analyzed using Illumina amplicon sequencing of the V4 region of the 16S rRNA gene as previously described^41^ (see Supplementary Methods).

### Microbiome sequence analysis

#### Alpha diversity

Alpha diversity comparisons were calculated within QIIME (v 1.8) for Shannon (*H*’) and^42^ Phylogenetic Diversity (PD) Whole Tree^43^ metrics on subsampled Operating Taxonomic Unit (OTU) tables (rarefied) to a depth of 28,927 sequences for fecal and 2,000 sequences for nasal samples. A non-parametric t-test using 1000 Monte Carlo permutations and Benjamini-Hochberg false discovery rate (FDR) correction was used to compare alpha diversity at each study time point.

Probiotic/placebo treatment differences in alpha diversity (PD whole tree) over time were investigated using SAS^®^ System for Windows, version 9.3 (SAS Institute Inc., Cary, NC, USA) with a repeated measures analysis of covariance (RM-ANCOVA) model separately for change from study day (D) −28 and D0 in logarithmic values of alpha diversity. The models included fixed effects of treatment, time and D-28/D0 alpha diversity value as a baseline covariate. The interaction of treatment and time was also included. The connection of infection symptom scores with alpha diversity was investigated with similar models by first including the total symptom score divided in 2 categories by median to the model as a fixed effect instead of treatment. Geometric mean estimates for over time differences between symptom groups and estimates by time point for the symptom group differences were calculated from the same model with contrasts. In case the symptom effect was found significant, also treatment and treatment interaction with the symptom score and the interaction of symptom score and time point were tested. Additionally, cluster effect on the alpha diversity values over time was evaluated with a repeated measures analysis of variance (RM ANOVA). The model included alpha diversity in logarithmic scale as a response and the fixed effects of time-dependent cluster and time point. Geometric means for over time cluster differences were calculated with contrasts from the same model. P-value < 0.05 was considered significant. Due to the explorative post hoc nature of the RM analyses, P-values were unadjusted.

#### Beta diversity

Beta diversity metrics were calculated within QIIME (v 1.8) using unweighted and weighted UniFrac metrics^44^ on rarefied OTU tables. Adonis (PERMANOVA test) from the R-vegan package within QIIME was used to determine the strength of sample clustering by study group (placebo vs probiotic at each time point), or cohort and cluster. The resulting distance matrices were visualized using principal coordinates analysis (PCoA) with the R (v. 3.4) *ggplot2* package^45^. Independent from the intervention and rhinovirus challenge, nasal samples from a cohort 1 (studied in the spring) clustered separately from cohorts 2 (studied in the spring) and 3 (studied in the autumn) (Supplementary Fig. 3) according to both unweighted UniFrac (PERMANOVA R^2^ = 13.1% of variation explained; P = 0.001) and weighted UniFrac (PERMANOVA R^2^ = 7.22% of variation explained; P = 0.001). This was attributed to low abundance OTUs, as reflected by the greater R^2^ for unweighted UniFrac and was not investigated further. In addition, there was an almost negligible but significant effect of study cohort on sample clustering by unweighted UniFrac (PERMANOVA R^2^ = 0.593% of variation explained; P = 0.001) and weighted UniFrac (PERMANOVA R^2^ = 1.41% of variation explained; P = 0.001) in the fecal sample analysis.

#### Microbial taxa discrimination

Taxa discrimination between treatment groups at each study time point was determined by the Kruskal-Wallis test within QIIME (v 1.8). *P* values were corrected for multiple testing by Benjamini-Hochberg false discovery rate (FDR) correction.

#### Clustering of nasal microbiota

Clustering of the samples into clusters was done based on the weighted UniFrac metric distance matrix including all samples from all time points. Unweighted pair-group method (UPGMA) with arithmetic averages was used for the clustering. The decision of the optimal number of clusters was made based on peak values in the pseudo F and pseudo t-squared values. The preliminary clustering revealed 2 outliers, which were samples that were clearly further away from all the other samples forming their own single sample clusters. These samples were removed from the final clustering. Within-subject changes in the cluster were evaluated descriptively with frequency tables.

#### Correlation analyses of nasal microbial clusters and alpha diversity with clinical data

The effect of treatment and infection status and gender on the cluster were investigated with separate logistic regression generalized estimating equation (GEE) models, where only the two most common clusters were included in the analysis. The frequencies by time point in the smaller clusters were not adequate for analysis. The treatment model included the cluster as the dependent variable, treatment and day, the interaction of treatment and day as predictor variables, and D-28 cluster as a baseline covariate. Probability to be clustered in the *Staphylococcus* cluster was modelled. Similar model was fitted for gender and infection, for the infection model the analysis population included only subjects challenged to the virus.

The effect of baseline cluster on infection symptoms (WURSS-21, total cold symptom, rhinorrhea, nasal obstruction, cough sneezing) was analyzed with a negative binomial GEE model by using the score variable as the dependent variable and D0 cluster and D0 daily score as predictor variables. Since two of the clusters were more uncommon with lower frequencies (<5 subjects per treatment group within the cluster), the four most common clusters (*Staph*, *Cor/All, Mor*, *Ps*/Mix) were included to the analysis. If the cluster was found to be a significant factor, treatment effect and the interaction of cluster and treatment were also introduced to the model. Due to the explorative post hoc nature of the analyses, p-values were unadjusted.

### Nasal inflammatory markers and viral titer

Nasal lavage samples were collected in saline. CXCL8 concentration was measured in nasal lavage using a commercially available ELISA assay (R&D Systems, Minneapolis, MN, USA) as previously described^46^. Other inflammatory markers were measured in nasal lavage fluid using a commercially available multiplex assay (Aushon BioSystems, Inc., Billerica, MA, USA) as previously described^24^.

The virologic methods used in this study have been previously described^24^. Briefly, nasal lavage specimens from study days 1–5 after challenge were cultured in duplicate cultures of MRC-5 and WI-38 cells. Neutralizing antibody titers were determined in acute and convalescent sera collected on Day 0 prior to challenge and on Day 21–28 after challenge. Volunteers with either positive viral cultures or seroconversion were considered infected. Viral titers were determined in the nasal wash specimens stored at −80 °C by culturing serial 10-fold dilutions in microtiter plates of MRC-5 cells as previously described^47^.

Change from D0 in inflammatory response values were evaluated with RM-ANCOVA models. Models included the change from D0 in logarithmic scale as a response, fixed effects of treatment, visit, D0 cluster, interaction of treatment*visit and D0 response value as a baseline covariate. In case a significant D0 cluster effect was detected, interaction of D0 cluster and visit was introduced to the model. Geometric mean estimates for over time differences between D0 clusters were calculated from the same model with contrasts. Similar model was fitted also for viral titer values (log). Only baseline covariate was not included to this model, since absolute (log) D1–D5 values were modelled instead of change. Due to the explorative post hoc nature of the analyses, p-values were unadjusted.

### Symptom scoring

Symptom scoring was done daily on each of the five days after virus challenge using WURSS-21 questionnaire^4,48^ that assesses both cold symptom severity and functional status with subjective score of 0 (no symptoms) to 7 (severe symptoms). The total cold symptom score reported here includes evaluations on rhinorrhea, nasal obstruction, sneezing, sore throat, cough, hoarseness, head congestion, chest congestion, and feeling tired.

## Electronic supplementary material

Supplementary Information
