# Supplementary material for: Nasal microbiota clusters associate with inflammatory response, viral load, and symptom severity in experimental rhinovirus challenge
Source: Sci Rep. 2018 Jul 30;8:11411. doi: 10.1038/s41598-018-29793-w (PMC6065324; doi:10.1038/s41598-018-29793-w)
Supplement: Supplementary file 1 — Supplementary Information [file 41598_2018_29793_MOESM1_ESM.pdf]

Title:

Nasal microbiota clusters associate with inflammatory response, viral load, and symptom severity in experimental rhinovirus challenge

Authors:

Markus J. Lehtinen[1*], Ashley A. Hibberd[2], Sofia Männikkö[3], Nicolas Yeung[1], Tommi Kauko[3], Sofia Forssten[1], Liisa Lehtoranta[1], Sampo J. Lahtinen[1], Buffy Stahl[2], Anna Lyra[1], and Ronald B. Turner[4*].

Affiliations:

1) Global Health & Nutrition Science, DuPont Nutrition and Health, Sokeritehtaantie 20, FI-02460 Kantvik, Finland

2) Genomics and Microbiome Science, DuPont Nutrition and Health, 3329 Agriculture Drive, Madison, WI 53716 US

3) 4Pharma Ltd., ElectroCity, Tykistökatu 4D, FI-20520 Turku, Finland

4) University of Virginia School of Medicine, P.O. Box 800386, Barringer Building, Room 4441, Hospital Drive, Charlottesville, VA 22908, US

Corresponding author:

Markus J. Lehtinen

Global Health & Nutrition Science, Danisco Sweeteners Oy, Sokeritehtaantie 20, FI-02460 Kantvik, Finland

E-mail: markus.lehtinen@dupont.com

Tel: +358 40 139 7813

Supplementary Information

Supplementary Methods

DNA extraction from fecal and nasal samples

Microbial DNA was extracted from nasal swabs and purified with an automated MagMAX™ Sample Preparation System (Life Technologies, Halle, Belgium), by using the MagMAX™ Total Nucleic Acid Isolation Kit, AM1840 (ThermoFisher Scientific OY, Vantaa, Finland) according to the manufacturer's instructions with some modifications. The swabs were transferred into tubes to which 700 µl of PBS was added. The tubes were vortexed (45 s). The swabs were placed upside in tubes that were centrifuged at 10 000 x g (5 min). The supernatant was removed and 175 µl of PBS was added. Lysis/binding solution (235 µl) and the sample suspension was added to the bead tube and vortexed 3 x 30 s (6800 rpm) with a Precellys bead beater (Bertin Instruments, ThermoFisher Scientific). Afterwards manufacturer's instructions were followed.

Microbial DNA from fecal samples was extracted as in Mukerji et al. [1]. Briefly, DNA was extracted and purified with a commercial kit MagMAX™ Total Nucleic Acid Isolation Kit, using the MagMAX™ Express-96 (Applied Biosystems, Bridgewater, NJ, USA), followed by PCR inhibitor removal in 96-well format silica columns (OneStep-96™ PCR Inhibitor Removal Kit, Zymo Research, Irvine, CA, USA). The DNA concentration was determined with the Qubit™ 2.0 Fluorometer and Qubit® dsDNA HS Assay Kit reagents (Life Technologies, Darmstadt, Germany).

Bl-04 quantitative PCR

Bl-04 was quantified with strain specific primers (forward primer 5'-CTTCCCAGAAGGCCGGGT-3' and reverse primer 5'-CGAGGCCACGGTGCTCATATAGA-3',

amplicon length 98 base pairs, annealing temperature 60˚C). The primers target a long-chain fatty acid CoA ligase gene present as a single copy per genome. The reactions were executed in a total volume of 15 µl: 1 x Fast SYBR Green Master mix (Applied Biosystems, Bridgewater, NJ, USA), 1 ng of template DNA and 1.5 pmol primers. The amplification and detection of DNA were performed with the 7500 Fast Real-Time PCR System (Applied Biosystems). For standard curves, 10-fold serial dilution from 10 ng to 100 fg of genomic DNA were used. Non-template and negative controls of *B. lactis* strain Bi-07 (genomic DNA 1ng) were used. Melt-curve assays were done after each run and the peaks of the samples were checked in reference to the target species (standard) peaks. No values detected below lowest standard or highest negative control, with a target-matching melt-curve, were accepted.

Microbiota sequencing and pre-processing

The microbiota composition was analyzed using high throughput amplicon sequencing as previously described [2]. Briefly, the V4 variable region of the 16S rRNA gene of Bacteria and Archaea was amplified in triplicate PCR with primers 515F (5'-GTGCCAGCMGCCGCGGTAA) and 806R (5'GGACTACHVGGGTWTCTAAT) PCR products were purified, normalized by DNA concentration and the amplicon pool was sequenced using Illumina MiSeq technology with 2x250 bp reads (University of Illinois, Urbana-Champaign, W. M. Keck Center for Comparative and Functional Genomics). The sequencing data was analyzed using the Quantitative Insights Into Microbial Ecology pipeline (QIIME v. 1.8) [3], and sequences were clustered into operational taxonomic units (OTUs) at 97% sequence similarity against the Greengenes database (v. 13.8) [4] using the default open reference clustering scheme in QIIME as described [5]. Taxa compositions are reported as relative abundance (% of total sequences) and were visualized using Prism (GraphPad Software, v. 7.0).[4,6-9]

Supplementary Figures

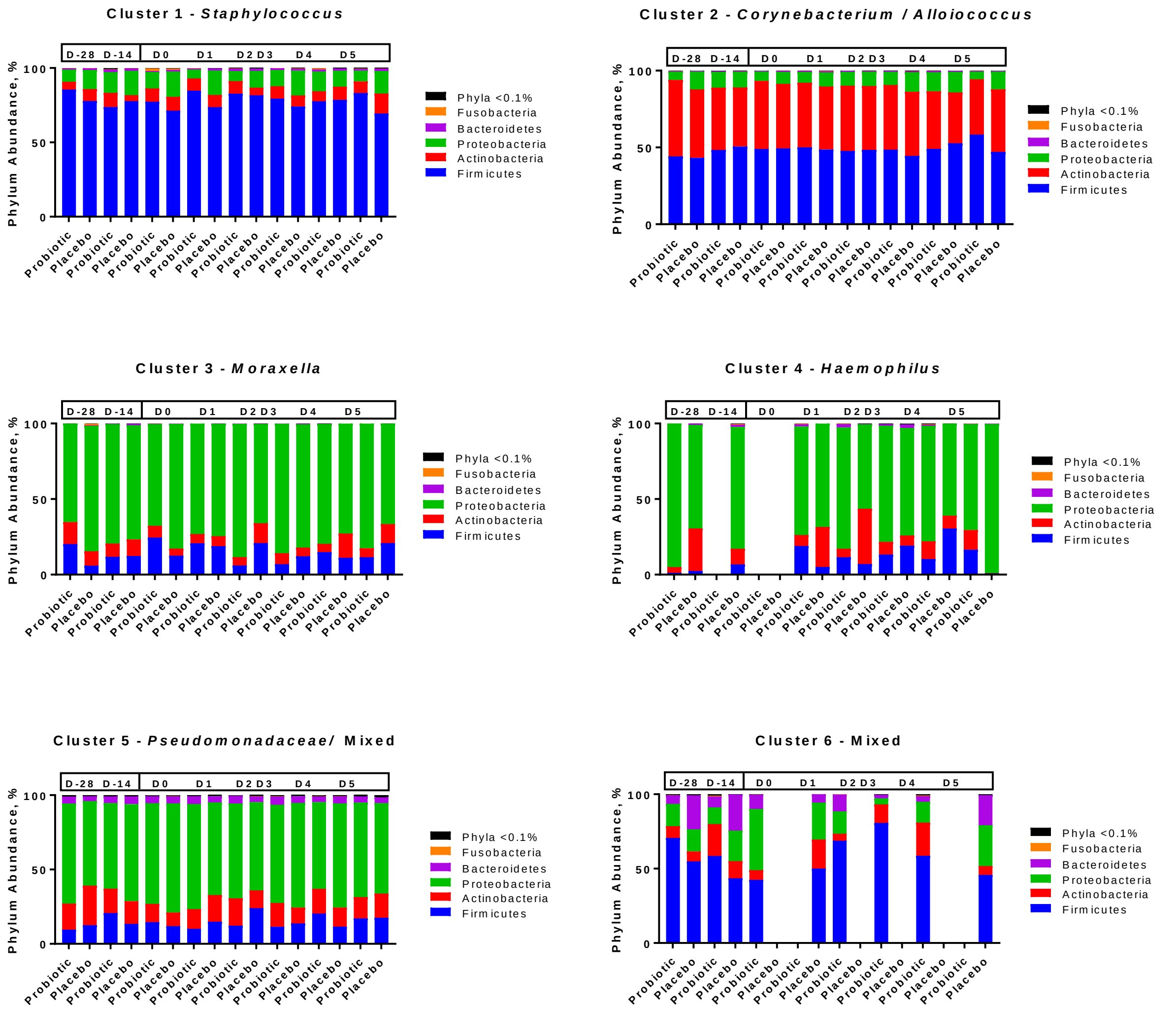

Supplementary Figure 1. The relative abundance (%) of bacterial phyla in the nasal microbiota in the six identified clusters.

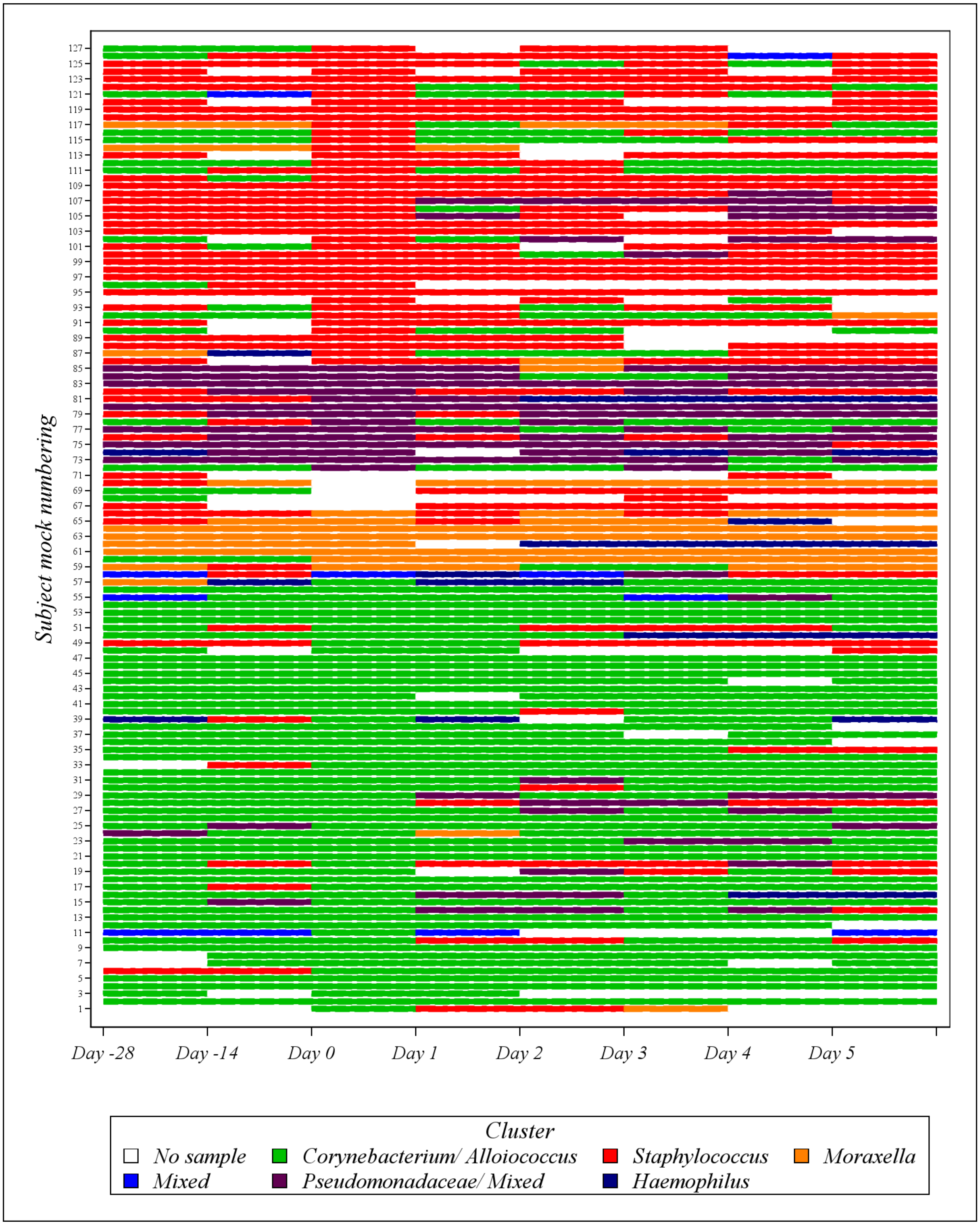

Supplementary Figure 2. Clusters assigned per subject and per timepoint. The subjects are in the order of D0 cluster assignments.

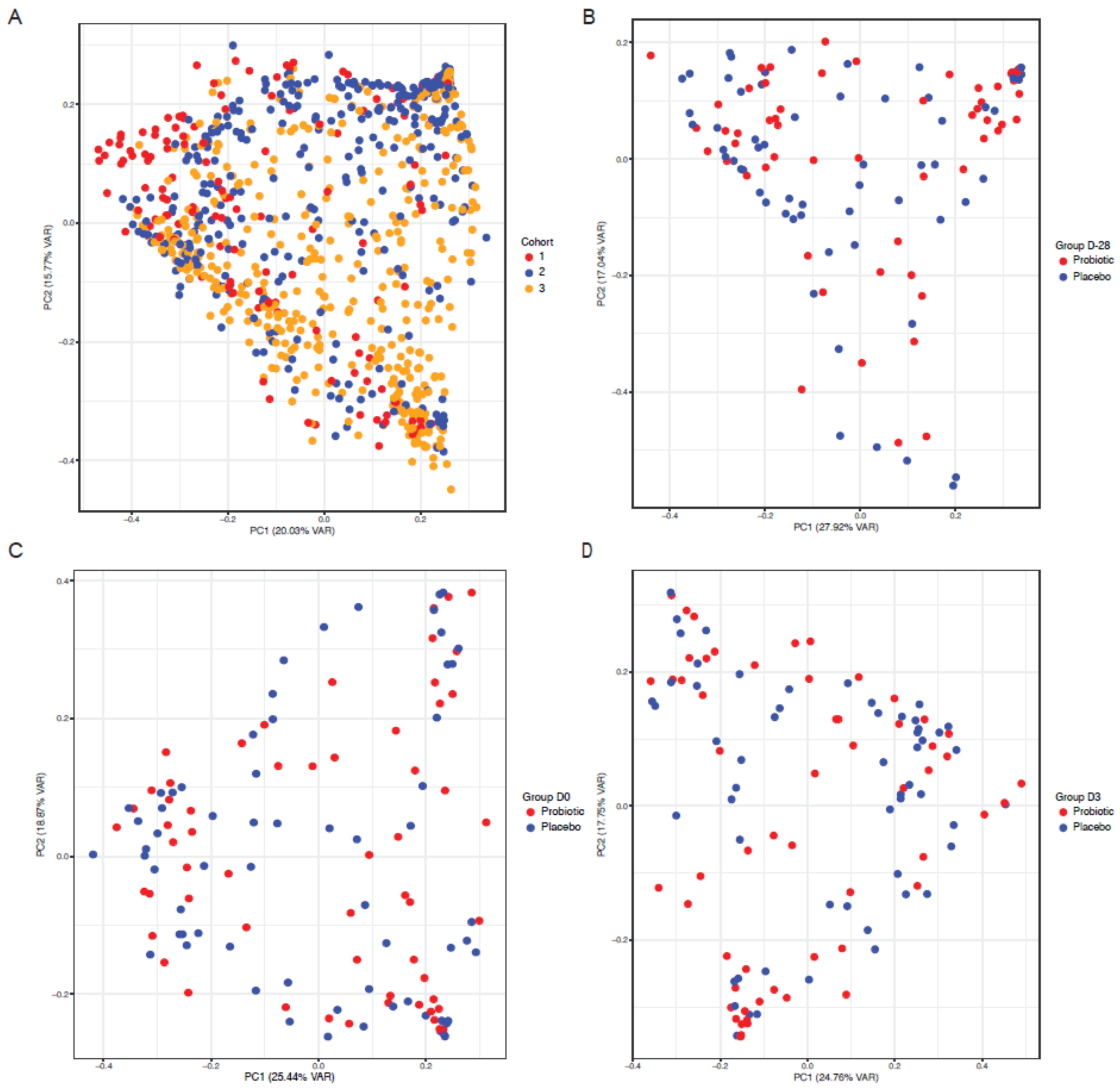

Supplementary Figure 3. Beta diversity in the nasal samples. Unweighted UniFrac PCoA plots. (a) between different cohorts, (b) between probiotic and placebo group on D-28 (baseline), (c)

between probiotic and placebo group on D0 (before infection), (d) between probiotic and placebo group on D3 (during infection).

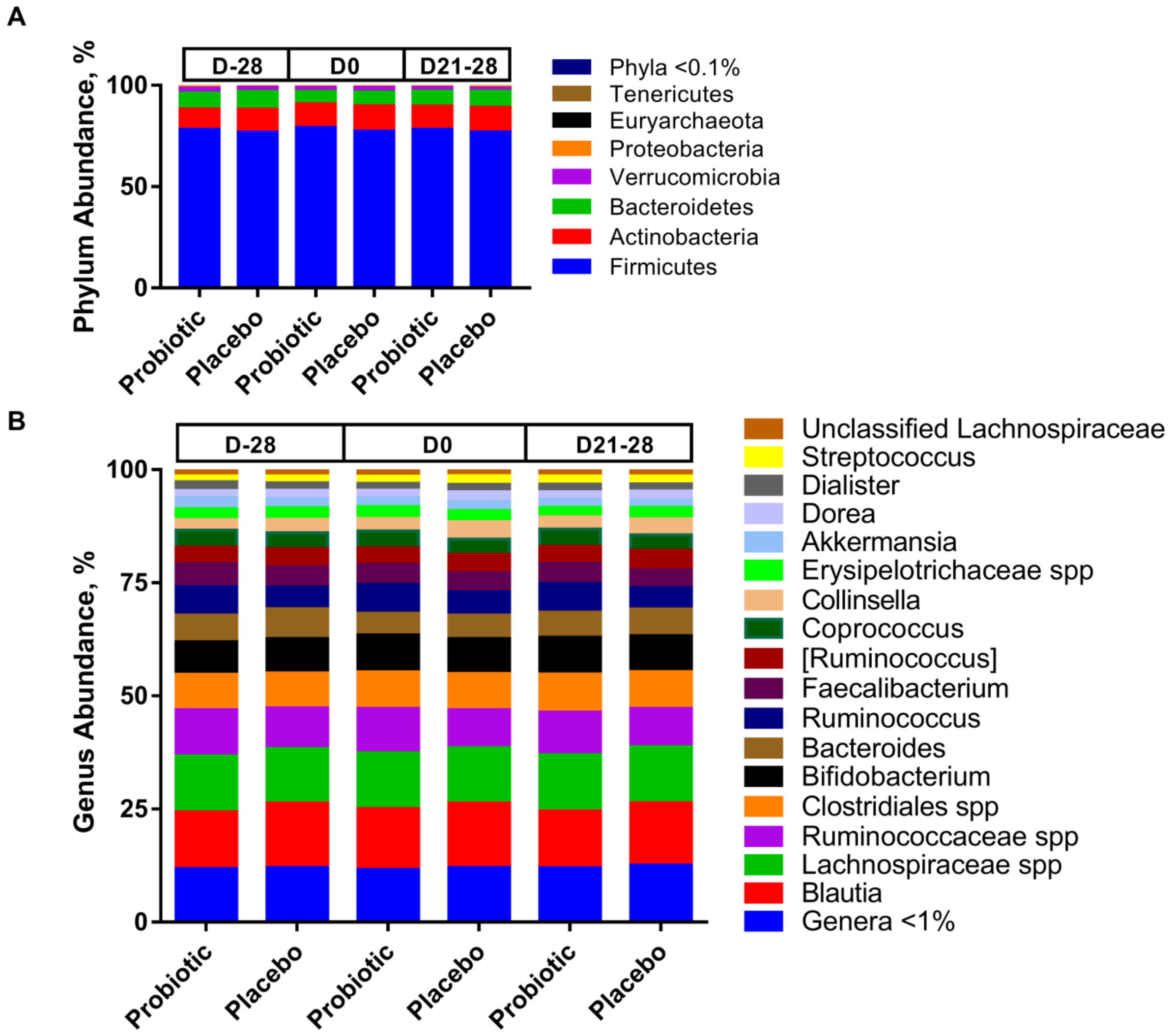

Supplementary Figure 4. The relative abundance (%) of bacterial a) phyla and b) genera in the fecal microbiota in the two study groups. The relative abundance of the samples showed that phyla and genera were representative of a typical intestinal microbiota. There was no statistical difference in the *Bifidobacterium* abundance in the probiotic group when compared to the placebo group respectively, at D-28 (7.2% vs 7.6%, P>0.1), D0 (8.2% vs 7.8%, P>0.1) or D28 (8.1% vs 7.9%, P>0.1) Kruskal-Wallis test with Benjamini-Hochberg false discovery rate correction).

SUPPLEMENTARY TABLES

Supplementary Table 1. Frequency of clusters by study day and treatment.

		Cluster name					
Day	Treatment	*Staphylococcus*	*Corynebacterium/ Alloiococcus*	*Moraxella*	*Haemophilus*	*Pseudomona daceae/* Mixed	Mixed
D-28	Probiotic, N (%)	21 (55 .3)	27 (43 .5)	4 (44 .4)	1 (50)	5 (62 .5)	2 (66 .7)
	Placebo, N (%)	17 (44 .7)	35 (56 .5)	5 (55 .6)	1 (50)	3 (37 .5)	1 (33 .3)
D-14	Probiotic, N (%)	21 (61 .8)	21 (38 .9)	3 (37 .5)		8 (61 .5)	1 (50)
	Placebo, N (%)	13 (38 .2)	33 (61 .1)	5 (62 .5)	2 (100)	5 (38 .5)	1 (50)
D0	Probiotic, N (%)	22 (52 .4)	24 (42 .1)	5 (62 .5)		8 (57 .1)	1 (100)
	Placebo, N (%)	20 (47 .6)	33 (57 .9)	3 (37 .5)		6 (42 .9)	
D1	Probiotic, N (%)	18 (50)	26 (46 .4)	3 (37 .5)	2 (66 .7)	8 (61 .5)	
	Placebo, N (%)	18 (50)	30 (53 .6)	5 (62 .5)	1 (33 .3)	5 (38 .5)	1 (100)
D2	Probiotic, N (%)	18 (51 .4)	24 (46 .2)	6 (60)	2 (66 .7)	7 (43 .8)	1 (100)
	Placebo, N (%)	17 (48 .6)	28 (53 .8)	4 (40)	1 (33 .3)	9 (56 .3)	
D3	Probiotic, N (%)	22 (64 .7)	20 (38 .5)	3 (37 .5)	3 (75)	8 (57 .1)	1 (100)
	Placebo, N (%)	12 (35 .3)	32 (61 .5)	5 (62 .5)	1 (25)	6 (42 .9)	
D4	Probiotic, N (%)	16 (51 .6)	21 (42)	4 (57 .1)	4 (80)	11 (57 .9)	1 (100)
	Placebo, N (%)	15 (48 .4)	29 (58)	3 (42 .9)	1 (20)	8 (42 .1)	
D5	Probiotic, N (%)	20 (52 .6)	20 (43 .5)	4 (50)	5 (83 .3)	9 (69 .2)	
	Placebo, N (%)	18 (47 .4)	26 (56 .5)	4 (50)	1 (16 .7)	4 (30 .8)	1 (100)

Supplementary Table 2. Cluster frequencies per cohort over all the time points and at D0.

	Cluster name					
Cohort	*Staph*	*Cor/All*	*Mor*	*Hae*	*Ps*/Mix	Mix
1, N (%)	26 (18.1)	92 (63.9)	19 (13.2)	7 (4.9)		
2, N (%)	143 (41.4)	150 (43.5)	39 (11.3)	8 (2.3)	1 (0.3)	4 (1.2)
3, N (%)	119 (27.0)	187 (42.5)	8 (1.8)	10 (2.3)	109 (24.8)	7 (1.6)
Cohort D0						
1, N (%)	4 (21.1)	12 (63.2)	3 (15.8)			
2, N (%)	23 (51.1)	18 (47.4)	4 (8.9)			
3, N (%)	15 (25.9)	24 (42.9)	1 (1.7)		14 (24.1)	1 (1.7)

Supplementary Table 3. Comparison of nasal sample alpha-diversity (within sample diversity) by time and between treatment groups (placebo vs. probiotic).

			Phylogenetic Diversity (Faith's metric)			Shannon Diversity Index		
Day	Treatment	N	Mean	SD	FDR P value[a]	Mean	SD	FDR P value[a]
D-28	Probiotic	60	9.063	4.765	0.535	2.537	1.509	0.891
	Placebo	62	8.581	4.309		2.501	1.347	
D-14	Probiotic	54	10.889	4.757	0.284	2.988	1.578	0.221
	Placebo	59	9.878	5.016		2.621	1.516	
D0	Probiotic	60	9.935	4.891	0.201	2.709	1.554	0.527
	Placebo	63	8.947	3.677		2.537	1.370	
D1	Probiotic	57	9.330	4.700	0.890	2.621	1.525	0.883
	Placebo	60	9.451	4.379		2.656	1.333	
D2	Probiotic	58	9.661	4.306	0.913	2.656	1.544	0.572
	Placebo	59	9.752	4.648		2.820	1.454	
D3	Probiotic	57	9.412	4.948	0.944	2.621	1.639	0.684
	Placebo	56	9.467	4.519		2.733	1.376	
D4	Probiotic	57	10.484	4.937	0.446	2.986	1.664	0.640
	Placebo	57	9.751	4.841		2.836	1.507	
D5	Probiotic	58	9.488	4.393	0.720	2.546	1.500	0.442
	Placebo	54	9.170	4.337		2.758	1.325	

[a] Non-parametric t-test using 1000 Monte Carlo permutations and Benjamini-Hochberg false discovery rate (FDR) correction

Supplementary Table 4. Comparison of nasal beta diversity (between sample dissimilarity) by cluster, cohort, and between treatment groups at each study time point (placebo vs probiotic).

Parameter	Unweighted UniFrac PERMANOVA R^2	Unweighted UniFrac P value[a]	Weighted UniFrac PERMANOVA R^2	Weighted UniFrac P value[a]
Cluster	0.0508	0.001	0.4889	<0.001
Cohort	0.1310	0.001	0.0722	0.001
Group D-28	0.0072	0.630	0.0097	0.291
Group D-14	0.0089	0.414	0.0118	0.246
Group D0	0.0068	0.699	0.0020	0.959
Group D1	0.0046	0.998	0.0053	0.695
Group D2	0.0068	0.748	0.0026	0.936
Group D3	0.0065	0.860	0.0095	0.379
Group D4	0.0071	0.686	0.0049	0.735
Group D5	0.0056	0.977	0.0096	0.396

[a] PERMANOVA test, Adonis R-*vegan* package

Supplementary Table 5. Comparison of fecal sample alpha-diversity (within sample diversity) by time and between treatment groups (placebo vs probiotic). The alpha diversity was lower according to Shannon diversity index at all analyzed time-points for the fecal microbiota in the placebo group, including the baseline (FDR P= 0.014 (D-28), FDR P=0.016 (D0) and FDR P=0.024 (D28)).

			Phylogenetic Diversity (Faith's metric)			Shannon Diversity Index		
Day	Treatment	N	Mean	SD	FDR P value[a]	Mean	SD	FDR P value[a]
D-28	Probiotic	70	87.203	14.925	0.035	7.328	0.438	0.014
	Placebo	72	81.636	17.604		7.105	0.578	
D0	Probiotic	72	86.500	16.652	0.121	7.266	0.423	0.016
	Placebo	79	82.100	17.050		7.063	0.568	
D28	Probiotic	73	86.701	16.558	0.075	7.320	0.491	0.024
	Placebo	78	81.845	16.759		7.124	0.553	

[a] Non-parametric t-test using 1000 Monte Carlo permutations and Benjamini-Hochberg false discovery rate (FDR) correction

Supplementary Table 6. Comparison of fecal beta diversity (between sample dissimilarity) by cohort, and between treatment groups at each study time point (placebo vs probiotic).

Parameter	Unweighted UniFrac PERMANOVA R^2	Unweighted UniFrac P value[a]	Weighted UniFrac PERMANOVA R^2	Weighted UniFrac P value[a]
Cohort	0.0059	0.001	0.0141	0.001
Group D-28	0.0072	0.630	0.0097	0.291
Group D0	0.0068	0.699	0.0020	0.959
Group D28	0.0056	0.977	0.0096	0.396

[a] PERMANOVA test, Adonis R-*vegan* package

Supplementary Table 7. Demographics shown for subjects in nasal and fecal microbiome analyses.

	Nasal swabs			Fecal samples		
	Probiotic N (%)	Placebo N (%)	P-value	Probiotic N (%)	Placebo N (%)	P-Value
Gender						
Male	23 (38%)	29 (44%)	0.475[a]	27 (37%)	33 (42%)	0.546[a]
Female	38 (62%)	37 (56%)		46 (63%)	46 (58%)	
Race						
Asian	3 (5%)	9 (14%)	0.138[b]	3 (4%)	10 (13%)	0.094[b]
Black	6 (10%)	2 (3%)		6 (8%)	2 (3%)	
Other	2 (3%)	1 (2%)		2 (3%)	1 (1%)	
White	50 (82%)	54 (82%)		62 (85%)	66 (84%)	
Ethnicity						
Hispanic	2 (3%)	3 (5%)	1.000[b]	3 (4%)	4 (5%)	1.000[b]
Non-hispanic	58 (95%)	62 (94%)		69 (95%)	74 (94%)	
Other	1 (2%)	1 (2%)		1 (1%)	1 (1%)	
Age (yrs, mean (SD))	22 (6)	22 (7)	0.766[c]	23 (6)	23 (7)	0.986[c]

[a] Chi-Square test

[b] Fisher's exact test

[c] T-test

References

1 Mukerji, P. *et al.* Safety evaluation of AB-LIFE((R)) (Lactobacillus plantarum CECT 7527, 7528 and 7529): Antibiotic resistance and 90-day repeated-dose study in rats. *Food Chem Toxicol* **92**, 117-128, doi:10.1016/j.fct.2016.03.018 (2016).

2 Caporaso, J. G. *et al.* Ultra-high-throughput microbial community analysis on the Illumina HiSeq and MiSeq platforms. *ISME J* **6**, 1621-1624, doi:10.1038/ismej.2012.8 (2012).

3 Caporaso, J. G. *et al.* QIIME allows analysis of high-throughput community sequencing data. *Nat Methods* **7**, 335-336, doi:10.1038/nmeth.f.303 (2010).

4 DeSantis, T. Z. *et al.* Greengenes, a chimera-checked 16S rRNA gene database and workbench compatible with ARB. *Appl Environ Microbiol* **72**, 5069-5072, doi:10.1128/AEM.03006-05 (2006).

5 Ghulam, S. R. *et al.* Polydextrose changes the gut microbiome and attenuates fasting triglyceride and cholesterol levels in Western diet fed mice. *Scientific Reports* **7** (2017).

6 Aronesty, E. Command-line tools for processing biological sequence data. (2011).

7 Edgar, R. C. Search and clustering orders of magnitude faster than BLAST. *Bioinformatics* **26**, 2460-2461, doi:10.1093/bioinformatics/btq461 (2010).

8 Caporaso, J. G. *et al.* PyNAST: a flexible tool for aligning sequences to a template alignment. *Bioinformatics* **26**, 266-267, doi:10.1093/bioinformatics/btp636 (2010).

9 Price, M. N., Dehal, P. S. & Arkin, A. P. FastTree 2--approximately maximum-likelihood trees for large alignments. *PLoS One* **5**, e9490, doi:10.1371/journal.pone.0009490 (2010).
